# BCL-XL and MCL-1 are the key BCL-2 family proteins in melanoma cell survival

**DOI:** 10.1038/s41419-019-1568-3

**Published:** 2019-04-24

**Authors:** Erinna F. Lee, Tiffany J. Harris, Sharon Tran, Marco Evangelista, Surein Arulananda, Thomas John, Celeste Ramnac, Chloe Hobbs, Haoran Zhu, Gency Gunasingh, David Segal, Andreas Behren, Jonathan Cebon, Alexander Dobrovic, John M. Mariadason, Andreas Strasser, Leona Rohrbeck, Nikolas K. Haass, Marco J. Herold, W. Douglas Fairlie

**Affiliations:** 1 La Trobe Institute for Molecular Science, La Trobe University, Melbourne, VIC 3086 Australia; 2 Olivia Newton-John Cancer Research Institute, Heidelberg, VIC 3084 Australia; 3 School of Cancer Medicine, La Trobe University, Melbourne, VIC 3086 Australia; 4 The Walter and Eliza Hall Institute of Medical Research, Parkville, VIC 3052 Australia; 5 The University of Queensland, The University of Queensland Diamantina Institute, Translational Research Institute, Woolloongabba, Brisbane, QLD 4102 Australia; 6 Department of Medical Biology, University of Melbourne, Parkville, VIC 3052 Australia; 7 Present Address: Peter MacCallum Cancer Centre, Melbourne, VIC 3000 Australia; 8 Present Address: Karolinska Institute, Stockholm, Sweden

**Keywords:** Melanoma, Apoptosis

## Abstract

Malignant melanoma is one of the most difficult cancers to treat due to its resistance to chemotherapy. Despite recent successes with BRAF inhibitors and immune checkpoint inhibitors, many patients do not respond or become resistant to these drugs. Hence, alternative treatments are still required. Due to the importance of the BCL-2-regulated apoptosis pathway in cancer development and drug resistance, it is of interest to establish which proteins are most important for melanoma cell survival, though the outcomes of previous studies have been conflicting. To conclusively address this question, we tested a panel of established and early passage patient-derived cell lines against several BH3-mimetic drugs designed to target individual or subsets of pro-survival BCL-2 proteins, alone and in combination, in both 2D and 3D cell cultures. None of the drugs demonstrated significant activity as single agents, though combinations targeting MCL-1 plus BCL-XL, and to a lesser extent BCL-2, showed considerable synergistic killing activity that was elicited *via* both BAX and BAK. Genetic deletion of BFL-1 in cell lines that express it at relatively high levels only had minor impact on BH3-mimetic drug sensitivity, suggesting it is not a critical pro-survival protein in melanoma. Combinations of MCL-1 inhibitors with BRAF inhibitors also caused only minimal additional melanoma cell killing over each drug alone, whilst combinations with the proteasome inhibitor bortezomib was more effective in multiple cell lines. Our data show for the first time that therapies targeting specific combinations of BCL-2 pro-survival proteins, namely MCL-1 plus BCL-XL and MCL-1 plus BCL-2, could have significant benefit for the treatment of melanoma.

## Introduction

Malignant melanoma is a difficult-to-treat cancer due to its capacity to metastasize and resist chemotherapy^1^. Patient outcomes have improved with the development of inhibitors targeting mutant BRAF found at high frequency in melanoma^2,3^. However, essentially all patients with *BRAF* mutations relapse, despite initially responding, as resistance occurs within 12 months^4,5^. Immunotherapy has also shown promise in melanoma patients. However, not all patients respond, side effects can be severe and acquired resistance remains a barrier to improving patient outcomes^5,6^. Hence, alternative treatments for melanoma are still required.

Defective apoptotic signaling is a hallmark of most cancers, including melanoma^1,7^, and contributes to therapeutic resistance. Intrinsic apoptosis is regulated by the BCL-2 protein family which includes pro-survival and pro-apoptotic subgroups^8^. The pro-survival members, BCL-2, BCL-XL, BCL-W, MCL-1 and BFL-1, have all been implicated in melanoma survival and chemoresistance.

Small molecule antagonists of the BCL-2 pro-survival proteins have now been developed^9^. These “BH3-mimetic” compounds engage their targets similarly to the natural pro-apoptotic ligands, the BH3-only proteins. The first-in-class BH3-mimetic was ABT-737 that binds BCL-2, BCL-XL and BCL-W with high affinity^10^. This compound, and its orally bioavailable analog ABT-263^11^, have been tested on melanoma cell lines in vitro and in vivo. Generally, their efficacy is poor^12–18^, implicating the pro-survival proteins not targeted (i.e., MCL-1 and/or BFL-1) in melanoma cell survival. Indeed, several studies showed improved cell killing when ABT-737 was combined with RNAi to reduce *Mcl-1* levels^14,17,19^, enforced expression of peptides that target MCL-1 (e.g., NOXA), or treatment with drugs that reduce MCL-1 and/or induce NOXA^13,14,16,17,20–22^. Similarly, co-targeting *Mcl-1* and *Bcl-x*_*L*_ by RNAi leads to greater killing than targeting either alone^19^. BFL-1 expression has been shown to be relatively high in melanoma^23–25^ and implicated in melanoma cell survival^17,19,23^ as *Bfl-1* knockdown enhances sensitivity to ABT-737 and other anti-cancer agents, though the effect is cell line dependent and in some cases minor^17,19,23,24^.

Recently, specific inhibitors of BCL-XL (WEHI-539, A1331852)^26,27^, BCL-2 (ABT-199/“Venetoclax”)^28^ and MCL-1 (A-1210477, S63845, AMG 176)^29–31^ were developed. Despite their high target affinities, these compounds generally have weak single agent efficacy in most tumors, except those of hematological origin. Of these compounds, only the MCL-1 inhibitor S63845 has been tested on melanoma cells and was generally ineffective as a single agent^29^. There are no small molecules targeting BFL-1, though a cell-penetrating BFL-1-selective peptide showed some activity in BFL-1-expressing melanoma cells^32^.

More recently, BH3-mimetic combinations were shown to act synergistically in hematological and some solid tumors^30,33–35^. In this paper, we tested the most potent BH3-mimetics in a panel of melanoma cell lines. Our studies showed that MCL-1 and BCL-XL must be co-targeted to achieve the most effective melanoma cell killing, though co-operativity was also observed by co-targeting MCL-1 and BCL-2. Using genetic approaches, we also demonstrated only a minor role for BFL-1 in melanoma cell responses to BH3-mimetics.

## Materials and methods

### Compounds

BH3-mimetic drugs (ABT-263, ABT-199, A1331852, S63845), PLX4032 and bortezomib were purchased from Selleckchem. Q-VD-OPh was from MP Biomedicals Australasia.

### Cell culture

Melanoma cell lines were cultured in RPMI medium (Gibco) supplemented with 10% (v/v) heat-inactivated fetal bovine serum (Moregate), 2 mM Glutamax (Gibco), 100 U/ml penicillin/streptomycin (Gibco) at 37 °C in a humidified incubator with 5% CO_2_.

### CellTiter-Glo luminescent cell viability assay

Cells (1000 per well) were seeded into white 96-well plates, then 4 h later treated with dilutions of drugs alone or in combination. Cell viability was assessed after 24 or 72 h treatment using the CellTiter-Glo assay (Promega) according to the manufacturer’s instructions with luminescence measured on an Ensight Multimode plate reader (PerkinElmer). Results were normalized to the viability of cells treated with the highest % (v/v) of vehicle. EC_50_ values were calculated using nonlinear regression algorithms in Prism software (GraphPad) from the combined data of multiple separate assays. Synergy analysis was performed using Combenefit software with Bliss, Loewe and HSA models^36^.

### Tissue microarray and immunohistochemistry analysis

Tissue microarrays were prepared as described previously^37^. Antigen retrieval for BCL-XL and MCL-1 was performed by boiling in a microwave for 15 min in citrate buffer (pH 6.0) or for BCL-2, with EDTA buffer (pH 8.0). After blocking with Background Sniper blocking reagent (Biocare Medical), sections were incubated with antibodies to BCL-XL, MCL-1 or BCL-2 (Cell Signaling Technology) overnight at 4 °C. After incubation with secondary antibodies (Cell Signaling Technology), signals were detected using the DAB Chromogen signal staining kit (Cell Signaling Technology). Positive control tissues included testes (for BCL-XL) and tonsil (for MCL-1 and BCL-2).

To evaluate antibody staining, cancer cells with positive staining in the cytoplasm were counted and the percentage of positive tumor cells graded as: 0: none; 1: 1–5%; 2: 6–25%; 3: 26–50%; 4: 51–75% and 5: 76–100%. The intensity of staining was rated as: 0: none; 1: weak; 2: moderate; 3: intense. An H-score for each sample was calculated by multiplying the grading and intensity scores (range 0–15), then categorized as high (9–15) or low (as 0–8).

### FACS-based apoptosis assay

Cells (30,000 per well) were seeded in a 24-well plate then 24 h later treated with drugs alone or in combination (±50 μM Q-VD-OPh) for 24 h. Cells were then washed with binding buffer before staining with Annexin V-APC (BD Biosciences) and propidium iodide (Sigma). Cells were analyzed on a BD FACSCanto II flow cytometer (BD Biosciences). Data were analyzed using FlowJo software with the % viable cells (Annexin V negative/ propidium iodide negative) calculated relative to % viable cells cultured in vehicle.

### 3D spheroid assays

Spheroids were prepared as described^38–40^. Briefly, 200 μl of melanoma cell suspension (25,000 cells/ml) was overlaid on top of hard (1.5% w/v) agarose. Plates were incubated for 72 h until 3D spheroids had formed. For the A02 cell lines, spheroids were then embedded into a gel of 40% (w/v) bovine collagen I (Cultrex® Bovine Collagen I) and allowed to grow and invade into the collagen matrix. Spheroids (either on agarose without embedding or after embedding) were treated with drugs at the concentrations and times indicated. To stain dead cells, DRAQ7 (Beckman Coulter Life Sciences) was added to a final concentration of 1.5 μM. Spheroids were imaged using an IN Cell Analyzer 2200 (GE Healthcare Life Sciences)/Nikon ×4/0.20, Plan Apo, CFI/60 or an Olympus FV3000 Laser Scanning Confocal Microscope/Olympus UPLSAPO ×4 or ×20, as indicated.

### Western blot analysis

Total protein extracts were prepared by lysing cells in lysis buffer (20 mM Tris-pH 7.4, 135 mM NaCl, 1.5 mM MgCl_2_, 1 mM EGTA, 10% (v/v) glycerol and 1% (v/v) Triton-X-100; Sigma-Aldrich) with protease inhibitor (Complete Mini, EDTA-free) for 1 h at 4 °C. Lysate supernatants equivalent to 200,000 cells were separated by SDS-PAGE (NuPAGE 4–12% Bis Tris gels, Invitrogen) before transferring to nitrocellulose and probing with antibodies to: MCL-1 (Walter and Eliza Hall Institute, clone 19C4-15), BCL-XL (BD Transduction Laboratory), BCL-2 (BD Biosciences), BFL-1 (Cell Signaling Technology), BAK (Sigma), BAX (BD Pharmingen), BIM (Walter and Eliza Hall Institute, clone 3C5), NOXA (Abcam) and β-actin (Sigma).

### CRISPR/Cas-9 gene deletion of BFL-1, BAX and BAK

Lentiviral particle production for Cas9 (with mCherry) and sgRNA (with CFP) expression was performed as described previously^41^. The *BFL-1* sgRNA has been characterized previously^29^ whilst the *BAX* and *BAK* sgRNA were designed using software provided at crispr.mit.edu. Virus-containing supernatants were used to infect cells at low MOI with Cas9-expressing lentivirus. mCherry^+^ cells were sorted (BD FACSAria III flow cytometer), subsequently re-infected with lentiviruses expressing sgRNA, then re-sorted for mCherry^+^/CFP^+^ cells. Expression of sgRNA was induced by treatment with doxycycline (1 μg/mL, Sigma) for at least 3 days prior to Western blotting to determine gene deletion efficiency, and treatment with BH3-mimetic drugs.

## Results

### Expression of BCL-2 proteins in melanoma cell lines

**Fig. 1:**
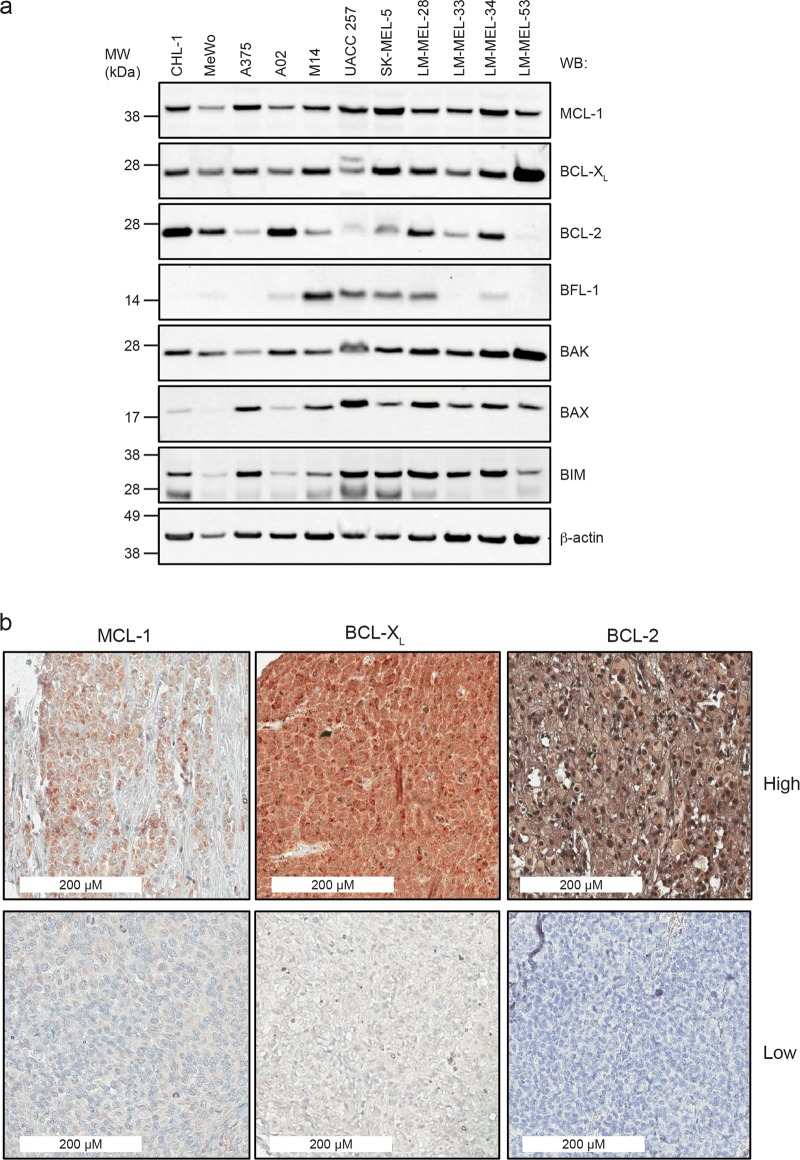
Expression levels of BCL-2 protein family members in melanoma cell lines and patient tissue. **a** Cell lysates from established, and patient-derived melanoma lines were analyzed by Western blot and probed for both pro-survival and pro-apoptotic BCL-2 family proteins. Blots were re-probed for β-actin as a loading control. **b** Representative samples from IHC analysis of melanoma tissue microarray showing examples of high and low expression of MCL-1, BCl-XL and BCL-2

We examined expression of key BCL-2 family proteins in a panel of melanoma cell lines, either long-established, or selected from the Ludwig Melbourne Melanoma (LM-MEL) panel derived from patients at the Austin Hospital (Melbourne, Australia)^42^. These included wild-type and mutant *BRAF* and *NRAS* lines (Supplementary Table 1). Both MCL-1 and BCL-XL were readily detectable in most lines (Fig. 1a). BCL-2 levels were more variable than MCL-1 and BCL-XL, whilst BFL-1 was detectable in ~ 50% of lines. Pro-apoptotic protein BAK was present in all cell lines, whilst BAX expression was more variable. The BH3-only protein BIM, essential for triggering apoptosis^18,43^, was also widely expressed. There was no obvious correlation between BCL-2 family protein expression and *BRAF/NRAS* status.

To confirm that key pro-survival proteins targeted by BH3-mimetics (BCL-2, BCL-XL, MCL-1) were expressed in more clinically-relevant patient-derived tissue, we performed immunohistochemistry on 127 biopsies from melanoma patients with Stage III/IV disease (Fig. 1b). High-level BCL-XL expression was observed in nearly all (126/127) samples. Interestingly, BCL-2 levels were also almost as uniformly high ( >90% of samples), whilst high MCL-1 was observed in ~50% of the samples. Hence, BCL-XL, BCL-2 and MCL-1 are all potential clinically-relevant targets in melanoma.

### Sensitivity of melanoma cell lines to BH3-mimetics as single agents

**Fig. 2:**
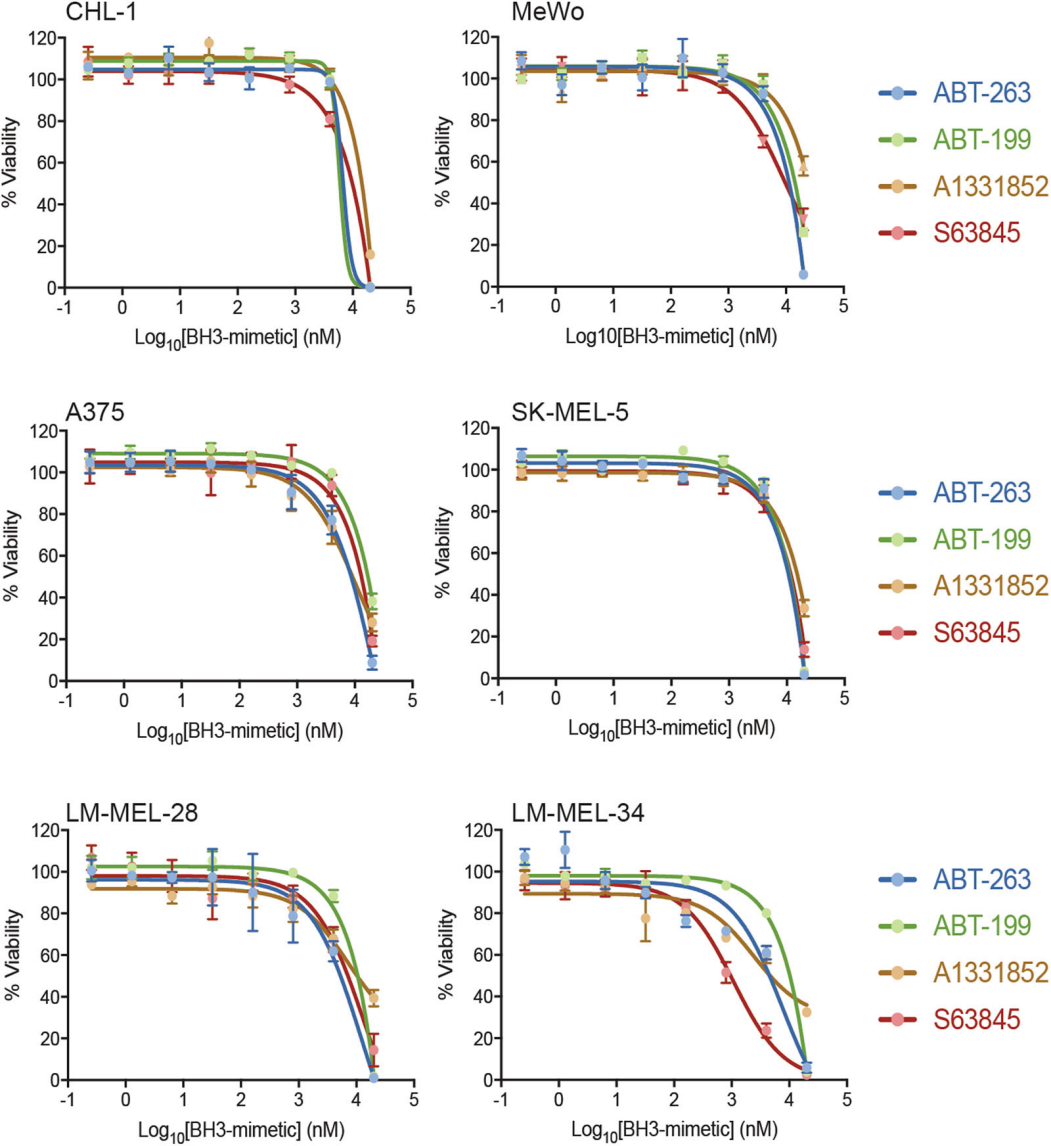
Melanoma cells are insensitive to BH3-mimetic drugs as single agents. Selective antagonism of BCL-XL (A1331852), BCL-2 (ABT-199) or MCL-1 (S63845), as well as co-targeting of BCL-XL, BCL-2 and BCL-W (ABT-263) fails to kill the majority of melanoma cells unless treated with high concentrations. Cell viability was determined after 24 h treatment by CellTiter-Glo luminescent assay. Data represent mean ± standard deviation from *N* = 3 separate assays. See Supplementary Figure 1 for corresponding data with other cell lines

We selected the most potent and/or clinically-relevant BH3-mimetics to target either individual or subsets of pro-survival proteins: ABT-263 (targeting BCL-2, BCL-XL, BCL-W), ABT-199 (targeting BCL-2), A1331852 (targeting BCL-XL) and S63845 (targeting MCL-1). Essentially all cell lines were resistant to the individual drugs, with most displaying EC_50_’s > 5 μM in CellTiter-Glo assays that measure ATP levels reflecting metabolically active cells and overall live cell numbers (Fig. 2, Supplementary Figure 1, Supplementary Table 2). These results with ABT-263 are consistent with reports using ABT-737 that has the same binding profile. As ABT-263 targets BCL-2, BCL-W and BCL-XL it was not surprising that ABT-199 and A1331852 also showed weak activity. Similarly, the weak activity of S63845 is consistent with another study using this compound on melanoma cells^29^. Hence, melanoma cells are not exclusively dependent on BCL-2, BCL-XL, or MCL-1 for survival, nor does co-targeting BCL-2, BCL-XL and BCL-W cause significant melanoma cell killing.

### Combination targeting of BCL-2 proteins reduces melanoma cell viability

**Fig. 3:**
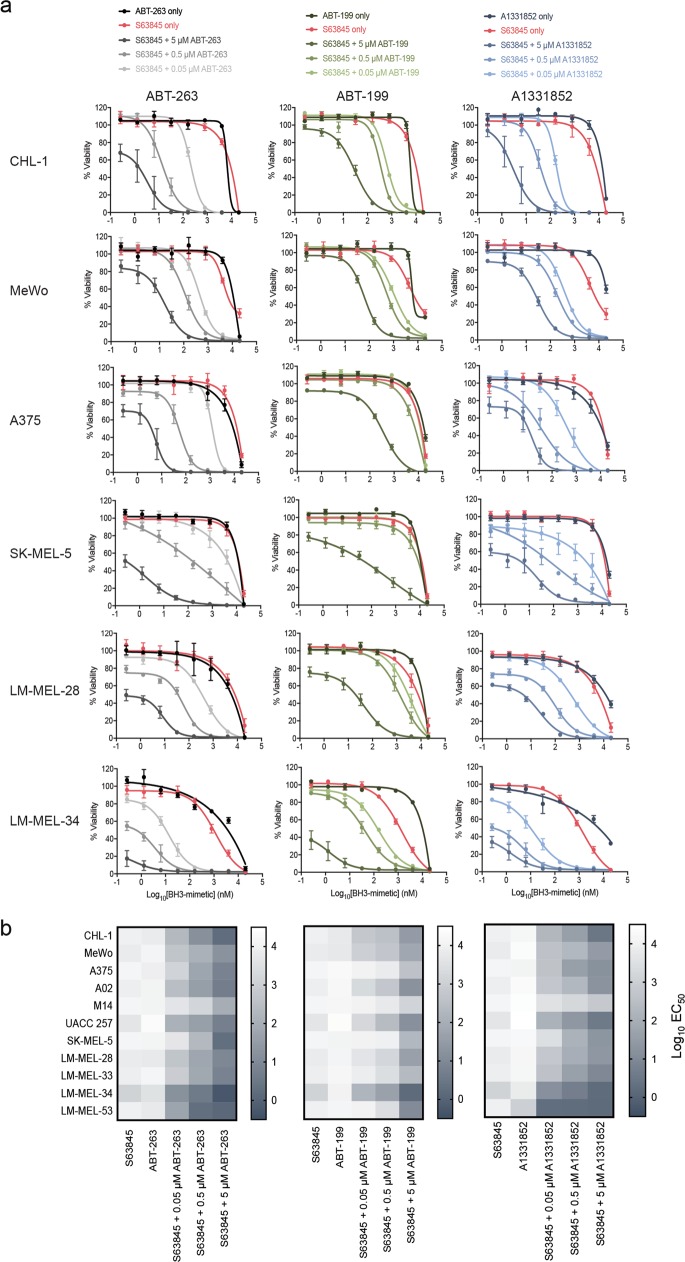
Co-antagonism of several BCL-2 pro-survival proteins has greater effect on cell viability than targeting each protein alone. **a** Antagonism of MCL-1 plus BCL-XL (S63845 + either A1331852 or ABT-263) is more effective than the combination targeting MCL-1 plus BCL-2 (S63845 + ABT-199) in all melanoma cell lines tested. Cell viability was determined after 24 h treatment by CellTiter-Glo luminescent assay. See Supplementary Figure 2 for data with other cell lines. Data represent mean ± standard deviation from *N* = 3–4 separate assays. **b** Heatmap of EC_50_ values for each cell line and drug combination. The S63845 plus ABT-199 combination was less effective than the other two combinations. See Supplementary Table 2 for actual EC_50_ values for all treatments

As MCL-1 is not targeted by ABT-263, BH3-mimetic combinations with S63845 could provide further insight into whether MCL-1 was critical for melanoma cell survival. Increased killing activity was observed when S63845 was titrated with a high dose (5 μM) of ABT-263, ABT-199 or A1331852, with EC_50_’s for S63845 decreasing from ~10 μM to <20 nM in most lines for the ABT-263 and A1331852 combinations (Fig. 3a, b and Supplementary Figure 2 and Supplementary Table 2). Moreover, combinations of S63845 with 500 nM of ABT-263 or A1331852 yielded sub-100 nM EC_50_’s and sub-1 μM EC_50_’s in many cell lines with doses as low as 50 nM. ABT-199 was less potent but also enhanced activity over S63845 alone when added at higher concentrations. The ABT-263 and A1331852 combination data closely mirrored each other, suggesting that the critical proteins for melanoma cell survival are MCL-1 and BCL-XL, although BCL-2 has some involvement. As potent activity was observed with combinations using drug concentrations where neither drug alone had a significant effect, this suggested that the responses were synergistic. This was confirmed, especially for the S63845 plus ABT-263/A1331852 combinations (Supplementary Figure 3a, b) using drug synergy analysis (i.e., Bliss, Loewe, Highest Single Agent)^36^

Patients are treated with multiple doses of BH3-mimetics over days/weeks. Whilst this is difficult to replicate in vitro, we nevertheless tested whether increased drug exposure times (i.e., 72 h) enhanced melanoma cell killing. We examined the S63845/ABT-263 combination on a subset of melanoma cell lines to target the greatest number of pro-survival proteins and thereby provide the best opportunity of an enhanced response. Increased (2–9-fold) killing was observed for each drug alone in some lines, though most remained resistant (EC_50_ > 2 μM) to single-agent treatment (Supplementary Figure 4). Increased activity (25–35-fold) was observed for the combination with ABT-263 at high concentration (5 μM), though only in two cell lines. At lower ABT-263 doses, the effects were minimal and in some cases, longer exposure was slightly less impressive than for the 24 h treatment, probably due to outgrowth of surviving cells after the initial response. These data suggest that increased exposure to BH3-mimetic combinations has some benefit, though most killing occurs within the first 24 h.

### Combination targeting of pro-survival BCL-2 proteins induces apoptosis

**Fig. 4:**
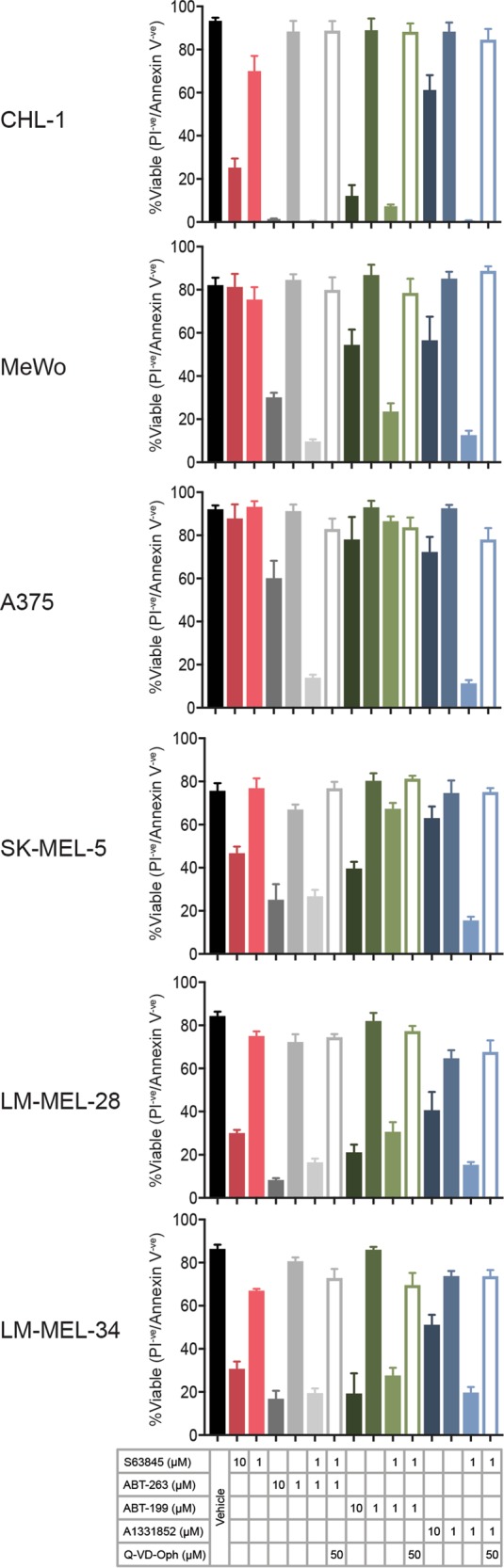
Combinations of BH3-mimetics induce apoptosis in melanoma cells as determined by Annexin V / propidium iodide staining. Co-targeting of MCL-1 plus BCL-XL is more effective than the co-targeting of MCL-1 plus BCL-2, consistent with the CellTiter-Glo assay data. For most cell lines, potent melanoma cell killing was observed after 24 h treatment when S63845 was combined with either ABT-263 or A1331852 at a concentration where both drugs had no major effect on their own (i.e., 1 μM). Melanoma cell killing was inhibited by the addition of the pan-caspase inhibitor Q-VD-OPh, consistent with induction of apoptosis. Data represent mean ± standard deviation from *N* = 3 separate assays

The CellTiter-Glo assay reflects live cell numbers which is influenced by both cell proliferation *and* cell survival. To ascertain that our results were consistent with cells undergoing apoptosis, we tested a subset of cell lines in FACS-based assays with Annexin V/propidium iodide staining. Each BH3-mimetic was tested individually (at 10 and 1 μM), and in combinations with each drug at 1 μM, and there was close correlation with the CellTiter-Glo assays. Significant effects with individual drugs were only observed at high concentrations (10 μM) (Fig. 4), whilst combinations of ABT-263 or A-1331852 (and to a lesser extent ABT-199) with S63845 were potent, despite each drug alone having minimal effect at 1 μM. Data for cell lines where there was a significantly weaker effect of the S63845/ABT-199 combination in the CellTiter-Glo assay (e.g., A375 and SK-MEL-5) was also reflected in the FACS-based assay. To further confirm apoptotic death, we demonstrated that the pan-caspase inhibitor (Q-VD-OPh) increased cell viability with drug combinations that otherwise induced killing (Fig. 4). Hence, BH3-mimetic combinations, particularly those targeting MCL-1 plus BCL-XL_,_ result in melanoma cell apoptosis.

### Combination targeting of BCL-2 proteins reduces melanoma cell viability in 3D spheroids

**Fig. 5:**
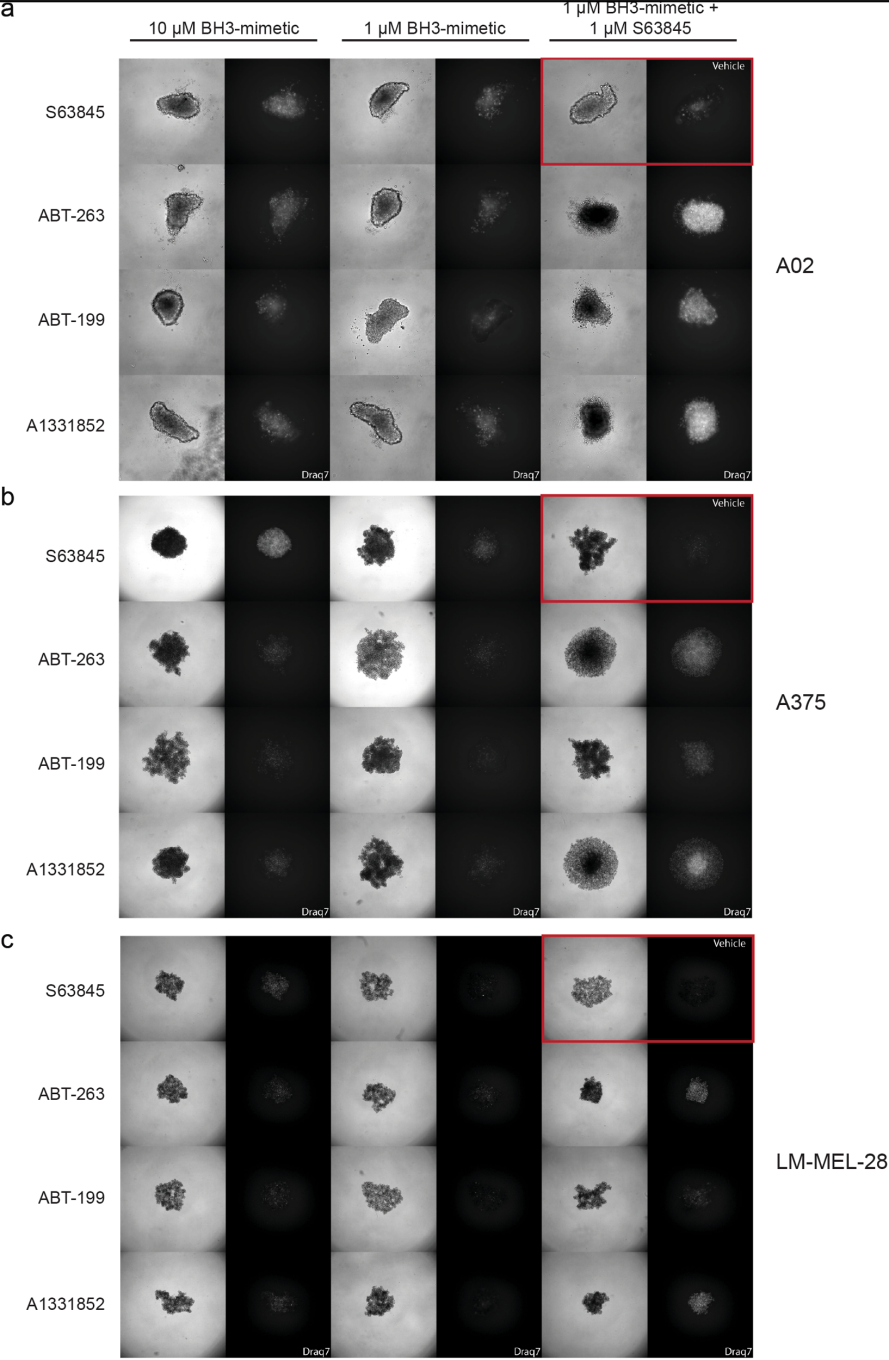
BH3-mimetics combinations are synergistic in 3D cultures. **a** A02, **b** A375, **c** LM-MEL-28 cells were allowed to form spheroids over 72 h and then treated for 24 h with drugs or combinations as indicated. Bright field images (left panel) and fluorescence images for DRAQ7 staining (right panel) are shown for each combination. The panel in the top right (red box) represents the vehicle only control. Spheroids were imaged with an IN Cell Analyzer 2200/Nikon ×4/0.20, Plan Apo, CFI/60. Note differences in DRAQ7 intensity and distribution. Data is representative from *N* = 3 independent experiments

We next expanded our studies to a more physiologically relevant system using melanoma spheroids that mimic the in vivo tumor architecture and microenvironment^44,45^. Of the three cell lines tested, A02 formed solid 3D spheroids while A375 and LM-MEL-28 formed more loosely organized clusters. Like the 2D cultures, treatment with 1 μM of each drug alone over 24 h resulted in little cell death (based on DRAQ7 staining intensity and distribution) in any cell line (Fig. 5). At 10 µM, S63845 had the strongest cell death-inducing effect as a single agent, though, cell line-dependent effects were also observed with the other compounds. More importantly, in all lines, S63845 with ABT-263/A1331852/ABT-199 (1 µM each) had a much greater effect than the drugs alone at 1 µM, though this was generally to a lesser extent for the S63845/ABT-199 combination (Fig. 5). We also tested A02 spheroids embedded into a collagen matrix which showed only slow invasion. In this context, 72 h drug combination treatment showed a stronger effect than single agent treatment, with the S63845/ABT-199 combination being the weakest, as reflected in the extent of full growth inhibition and invasion (Supplementary Figure 5). Hence, in 3D cultures, BH3-mimetic combinations targeting MCL-1 plus BCL-XL were most effective at killing melanoma cells, consistent with our 2D culture experiments, suggesting that this could be an effective targeting strategy in vivo.

### BAX and BAK are involved in BH3-mimetic killing of melanoma cells

To extend our mechanistic studies, we examined whether cell killing by BH3-mimetic combinations was mediated by BAX and/or BAK using LM-MEL-28 cells that expressed higher levels of BAX and BAK compared to other lines. *Bak* and *Bax* deletion was achieved using doxycyline-inducible CRISPR-Cas9 gene editing^41^. We screened multiple sgRNAs but only identified one for each gene that resulted in >50% deletion in a pooled cell population, with *Bax* deletion being more efficient (Supplementary Figure 6a). Cells expressing Cas9 plus *Bak* sgRNA without doxycycline treatment behaved similarly to parental LM-MEL-28 cells in response to BH3-mimetic combinations, with EC_50_ values within two-fold of each other (Supplementary Figure 6b). *Bak* deletion led to 2- > 20-fold reduction in cell killing by the S63845/BH3-mimetic combinations, with the greatest difference observed at the lowest concentration (50 nM) of ABT-263, ABT-199 or A1331852 (Supplementary Figure 6b). Cells transduced with the *Bax* sgRNA, but without doxycycline treatment, were slightly more sensitive (2–5-fold) than parental LM-MEL-28 cells for most drug combinations. However, *Bax* deletion had greater impact compared to *Bak* deletion, reducing sensitivity by 10- > 90-fold, and had greater effect at the higher drug doses (Supplementary Figure 6b). Hence, melanoma cell killing *via* BH3-mimetic combinations involves both BAX and BAK.

### Minor involvement of BFL-1 in melanoma cell survival

Our data with BH3-mimetics demonstrate the importance of targeting MCL-1 and BCL-XL to achieve potent killing of melanoma cells. However, studies have also shown that *Bfl-1* mRNA levels are relatively high in melanoma compared to other tumors^23,25^, and reducing BFL-1 levels by siRNA increases spontaneous cell killing and ABT-737 sensitivity, though this effect was small (~2-fold)^17,19,23^. To further investigate how BFL-1 levels influence melanoma cell sensitivity to BH3-mimetic combinations, we used validated sgRNAs^29,46^ to effectively delete *Bfl-1* in LM-MEL-28 and M14 cells (Supplementary Figure 7a) that have relatively high BFL-1 expression (Fig. 1a). The cells remained viable for several weeks with prolonged doxycycline treatment suggesting they were not solely dependent on BFL-1 for survival.

**Fig. 6:**
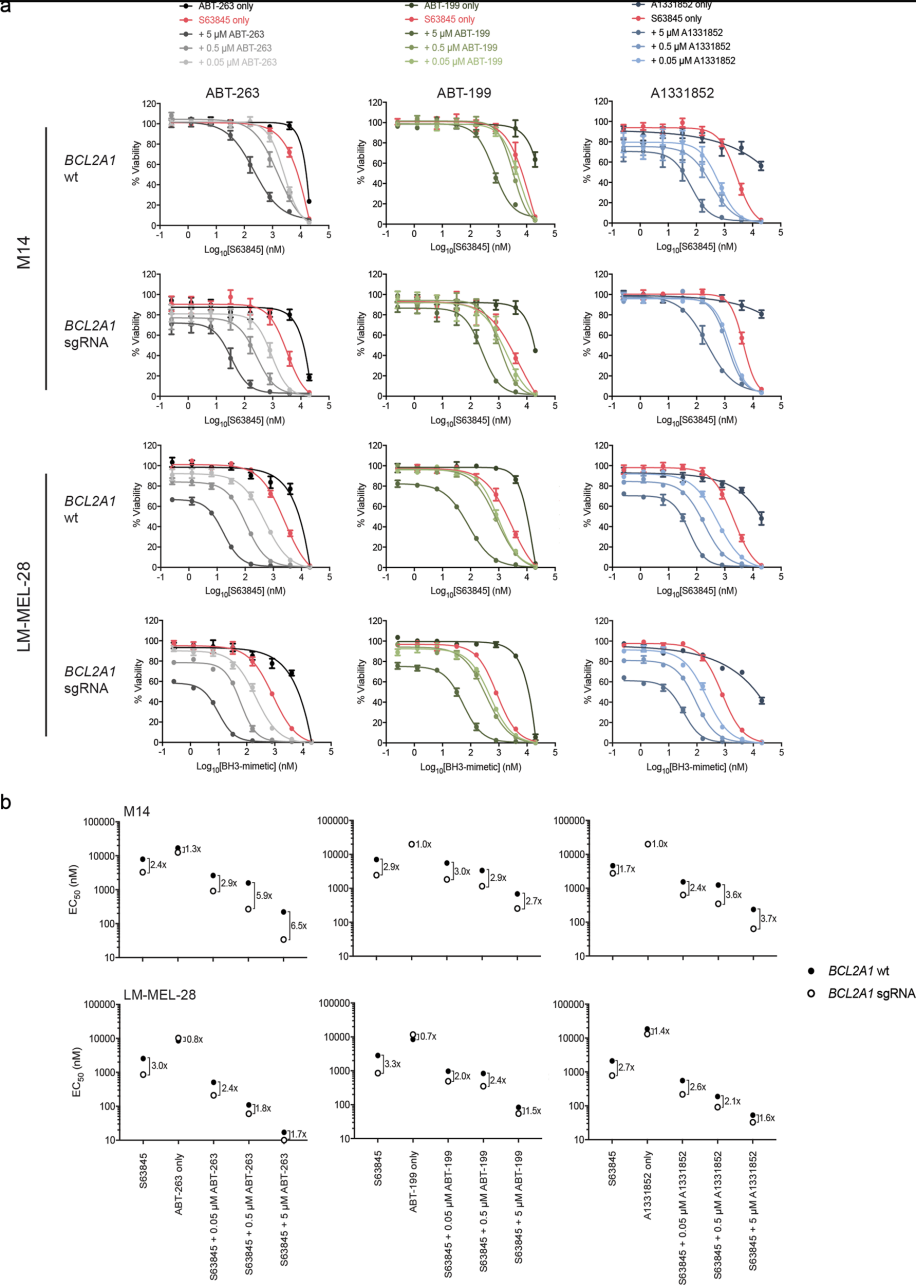
Deletion of BFL-1 together with co-antagonism of other pro-survival BCL-2 proteins has minor impact on melanoma cell killing. **a** Inhibition of both MCL-1 and BFL-1 in combination with either BCL-XL and BCL-2 antagonism increases melanoma cell killing in M14 cells, though not dramatically. **b** EC_50_ values for BH3-mimetic drug combinations in each BFL-1-deleted (*BCL2A1* sgRNA) and control (*BCL2A1* wt) cell line. The effect of BFL-1 deletion was slightly more significant in M14 cells compared to the LM-MEL-28 cells. Data represent mean ± standard deviation from *N* = 3 separate assays

We next examined whether *Bfl-1* deletion sensitized melanoma cells to BH3-mimetics. Both cell lines transduced with Cas9 and sgRNA lentiviruses, but without induction of *Bfl-1* deletion, behaved similarly to parental lines (<2-fold difference in EC_50_’s for all combinations). These lines remained relatively resistant to each BH3-mimetic alone, and *Bfl-1* deletion only had minor impact on sensitivity to various combinations (Fig. 6a, b and Supplementary Figure 7b). For M14 cells, EC_50_ values generally decreased <4-fold for most combinations, whilst for LM-MEL-28 cells, the effects were even more modest (1.5-2-fold increase in sensitivity). Hence, BFL-1 provides only a minor barrier to BH3-mimetic responses, even when relatively highly expressed.

### Co-treatment of melanoma cells with S63845 and other anti-cancer drugs

Several studies have examined the effect of combining ABT-737/ABT-263 with BRAF inhibitors^15,18,47^ or proteasome inhibitors in melanoma^13,14^. These combinations increased killing over treatment with either drug alone, though relatively high doses of the BH3-mimetics (>1 μM) were required for significant effects. Only one study has combined S63845 with a BRAF inhibitor, where enhanced sensitivity to a single combination dose was observed with one cell line compared to treatment with either drug alone^29^. To our knowledge no studies have examined S63845 and proteasomal inhibitor combinations.

**Fig. 7:**
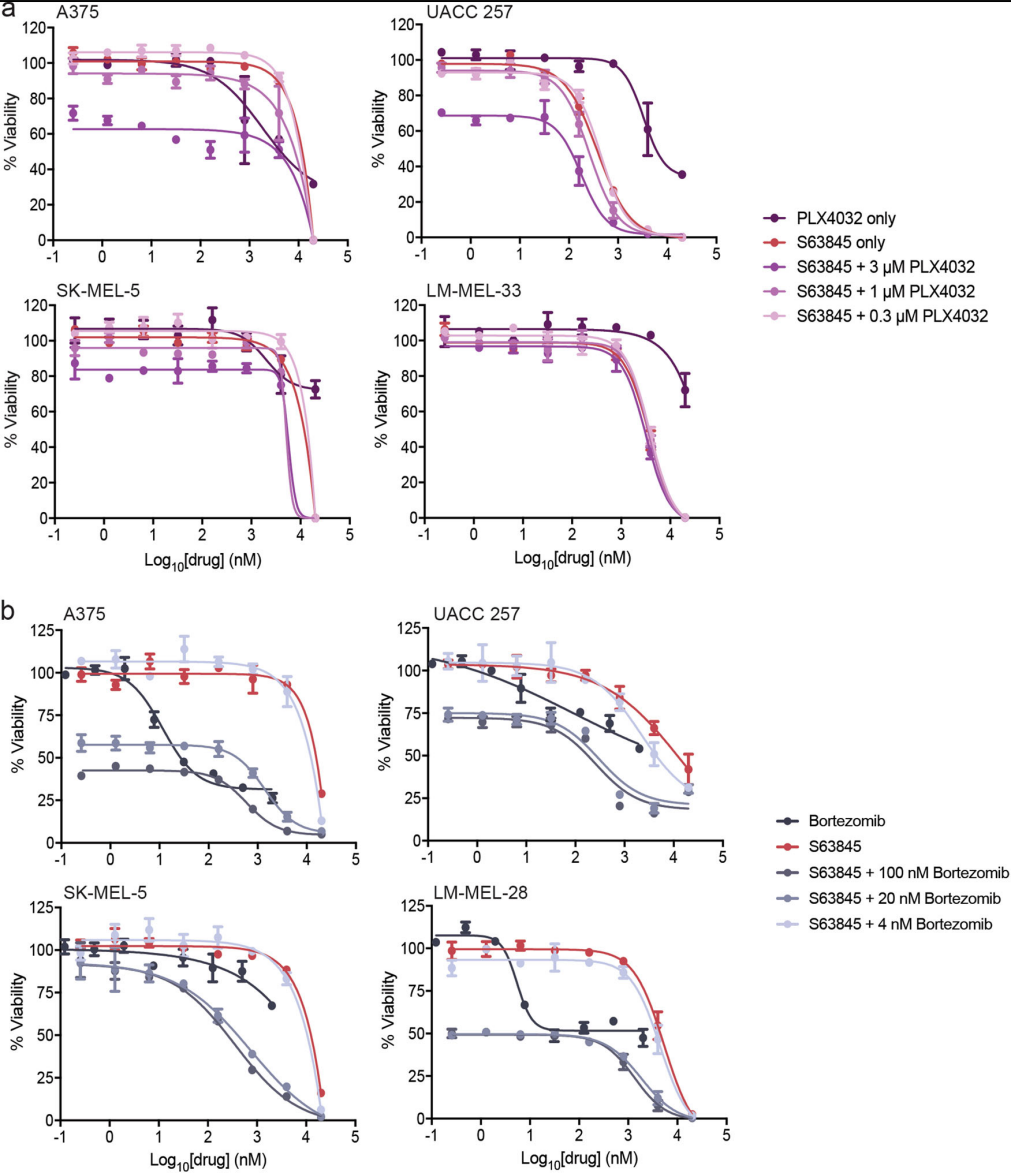
Co-treatment of melanoma cell lines with S63845 and either a BRAF inhibitor or a proteasome inhibitor has enhanced effect over treatment with each drug alone in certain melanoma cell lines. Melanoma cell lines were treated with (**a**), PLX4032 or (**b**), bortezomib, alone or in combination with S63845 for 72 h before analysis by CellTiter-Glo viability assay. Data represent mean ± standard deviation from *N* = 3 separate assays

We combined the BRAF inhibitor PLX4032 with S63845 in four BRAF mutant melanoma cell lines, but only observed enhanced killing over the individual drugs in the UACC 257 cells (Fig. 7a). Combinations of S63845 with the proteasome inhibitor, bortezomib, resulted in enhanced killing over treatment with either drug alone in UACC 257 and SK-MEL-5 cells, and this was observed at low doses (20 nM bortezomib with sub-1 μM S63845 concentrations), and the S63845 EC_50_ was reduced in all cell lines with 20 nM and 100 nM bortezomib (Fig. 7b). NOXA expression was induced at bortezomib concentrations where enhanced killing was observed (Supplementary Figure 8), consistent with its known mechanism-of-action^13,22^. Hence, these drug combinations can increase melanoma cell killing, though not in all cell lines tested.

## Discussion

The well-documented chemoresistance of melanoma has spurred multiple studies to identify the critical factor(s) that enable(s) tumor survival. These typically have focussed on the BCL-2 pro-survival proteins that are often over-expressed in tumor cells, and contribute to tumorigenesis and drug resistance^8,48^. Although these studies show that multiple pro-survival proteins are likely involved in melanoma survival, the combination(s) that must be targeted to achieve maximal cell killing was unknown. Moreover, no study has yet exploited the full suite of the most clinically relevant and well-characterized BH3-mimetics to ascertain the survival safeguards of melanoma cells.

Here, we showed that melanoma cells are protected by several BCL-2 pro-survival proteins, consistent with previous reports. More specifically, rigorous drug combination testing in both 2D and 3D cultures showed that maximal killing activity was achieved through co-targeting of MCL-1 and BCL-XL using combinations of S63845 with either A1331852 or ABT-263. This is consistent with the high-level expression of these proteins across essentially all of the cell lines examined.

Synergistic responses, albeit less pronounced, were also observed when MCL-1 and BCL-2 were co-targeted though this was more variable between cell lines. Notably, BCL-2 expression was also more variable (Fig. 1a), with weaker responses to the S63845/ABT-199 combination observed in lines with lower BCL-2 levels. This agrees with studies in other cancers showing high BCL-2 levels predict better responses to ABT-199^28^. Interestingly, high BCL-2 expression was observed in relatively more patient biopsies analyzed by immunohistochemistry compared to cell lines, suggesting that co-targeting of BCL-2 with MCL-1 could be more beneficial in patients than predicted by our cell line data alone. Similarly, the low MCL-1 levels in a significant proportion of patient samples could indicate patients might be more responsive to BCL-XL or BCL-2 inhibition alone than observed in cell lines. In some cell lines, longer treatment time (72 h *vs*. 24 h) was not as effective as shorter-term treatment at lower dose combinations. This also has clinically important implications as it suggests that resistant cell subpopulations could emerge leading to poor patient responses. Hence, further investigation into the basis of tumor cell heterogeneity and resistance mechanisms is warranted. Although BFL-1 has been implicated in melanoma cell survival, the results have varied between studies as to whether it is essential^17,19,23–25^. Our data demonstrated that cell lines expressing relatively high levels of BFL-1 can survive in its absence, and *Bfl-1* gene deletion only marginally influences BH3-mimetic sensitivity. This is consistent with other reports showing melanoma cell sensitivity to ABT-737 only increased ~2-fold following *Bfl-1* knock-down^17,19^.

As synergy was observed with combinations targeting MCL-1 plus *either* BCL-XL or BCL-2 (albeit to a lesser extent), our data support a model whereby a threshold level of pro-survival proteins must be antagonized to unleash sufficient pro-apoptotic molecules to activate apoptosis. Excess pro-survival proteins act as potential “sinks” for released pro-apoptotic molecules, hence, the need to inhibit multiple pro-survival proteins for potent killing in some tumors^34^. BCL-XL and MCL-1 are the critical guardians of BAK, whilst a wider complement of pro-survival proteins, including BCL-2 itself, restrain BAX^49–52^. Hence, it might be predicted that *Bax* deletion would impact more on ABT-199-mediated killing, whilst *Bak*-deleted cells would be most insensitive to the S63845/A1331852 combination. However, our knock-out studies showed that *both* BAX and BAK levels influence sensitivity to all BH3-mimetic combinations. A likely explanation for this is that both ABT-199 and A1331852 (as well as S63845 and ABT-263) can release pro-survival protein-bound BH3-only proteins, such as BIM, from their major direct targets^26,28,29^. As BIM, and some other BH3-only proteins, can directly activate *both* BAX and BAK, this could explain the involvement of both these proteins in responses to each drug combination. The less efficient deletion of *Bak* vs. *Bax* in our cells also should be considered, as complete deletion would likely have indicated a more important role for BAK than we demonstrated. Unfortunately, difficulty in deriving BAK-deleted clones prevented such assessment. Of note, the involvement of both BAX and BAK we observed contrasts with that observed in HCT116 colorectal cancer cells where the S63845/A1331852 combination results in BAX-dependent, but BAK-independent apoptosis^53^.

Recently, studies have shown that co-targeting of MCL-1 and BCL-XL could be effective in the treatment of breast^33,35^ and lung cancer^34^. Hence, a pattern is emerging that these are the more important pro-survival proteins in solid tumors. From a clinical viewpoint, co-targeting of MCL-1 plus BCL-XL is likely to be problematic due to on-target toxicities. Antagonism of BCL-XL alone results in thrombocytopenia^11,54^ whilst a recent report indicated that co-treatment of mice with S63845 plus A1331852 resulted in acute liver toxicity^34^.

Strategies to overcome these toxicities can be envisaged. For example, antibody-targeted nanoparticles allowing delivery of BH3-mimetics directly into tumor cells should be beneficial. Combinations of S63845 or A1331852 with other anti-cancer agents that downregulate BCL-XL or MCl-1 respectively in tumor *vs*. normal cells could also be effective. Similarly, co-treatment with drugs that induce BH3-only proteins (e.g., BIM, PUMA) to antagonize the non-targeted pro-survival BCL-2 family member, or directly activate BAX/BAK, could also have a similar effect. However, our studies combining S63845 with a BRAF inhibitor (that upregulates BIM and PUMA in melanoma cells^18^) only enhanced killing in one of four melanoma cell lines tested. Poor responses in the other cell lines could be due to multiple reasons, including insufficient BIM/PUMA upregulation to overcome the non-targeted pro-survival proteins present. Indeed, the cells that responded best to the S63845/PLX4032 combination were UACC 257 that had relatively lower BCL-2 and BCL-XL levels. We also tested S63845 with the proteasomal inhibitor bortezomib that has previously been shown to synergize with ABT-737^13,14^, probably due to its ability to upregulate NOXA. Interestingly, the S63845/bortezomib combination also enhanced melanoma cell killing over either drug alone in two cell lines, even at low drug concentrations, and the S63845 EC_50_ was reduced by bortezomib co-treatment in all cell lines. This enhanced killing activity correlated with increased NOXA expression. One possible reason for this synergy is that NOXA upregulation reduces the amount of “free” MCL-1 available to inhibit BAX/BAK, lowering the threshold for S63845 to antagonize additional MCL-1 molecules, thereby more efficiently inducing apoptosis. Recent studies have also shown that NOXA can directly activate BAK^55^, hence, MCL-1 inhibition by S63845 could make more of the upregulated NOXA available to induce apoptosis by this mechanism.

As the BH3-mimetic combinations with PLX-4032 and bortezomib only provided an advantage over both drugs alone in a subset of cell lines, such combinations may not be universally applicable, unlike pure BH3-mimetic-only combinations that synergize in all cell lines tested. Hence, one possible strategy to overcoming toxicities associated with targeting MCL-1 plus BCL-XL would be to exploit this potent synergy, and identify a therapeutic window where lower doses or treatment schedules might still be effective for killing tumor cells but sparing normal tissue. Moreover, reports showing MCL-1 and BCL-2 co-inhibition is tolerated in mice, and is synergistic in hematological malignancies, are starting to emerge^31,56,57^. Due to the synergy we observe with this combination in vitro in several melanoma cell lines, (and the apparent high BCL-2 expression in melanoma patient tissue), this could provide the most attractive avenue for future investigation of BH3-mimetics in melanoma.

Despite advances with BRAF inhibitors and immunotherapy, many patients still succumb to metastatic melanoma. Hence, alternative treatments are required. Here, we have conclusively identified MCL-1 and BCL-XL as the pro-survival proteins most critical for melanoma cell survival and co-targeting them results in synergistic killing. This provides a basis for future therapeutic approaches to melanoma treatment by directly targeting apoptotic pathways.

## Supplementary information

Supplementary Material
