# Supplementary material for: BCL-XL and MCL-1 are the key BCL-2 family proteins in melanoma cell survival
Source: Cell Death Dis. 2019 Apr 24;10(5):342. doi: 10.1038/s41419-019-1568-3 (PMC6482196; doi:10.1038/s41419-019-1568-3)

SUPPLEMENTARY FIGURES

SUPPLEMENTARY FIGURE LEGENDS

Supplementary Table 1

Cell lines used in this study and their BRAF /NRAS mutation status.

Supplementary Table 2

Summary of the EC_{50} values obtained from BH3-mimetic drug treatments used in this study (corresponding to data in Fig. 2 and Supplementary Fig. S1). Values were determined from CellTiter-Glo viability assays and represent the mean of n = 3-9 separate assays.

Supplementary Figure 1. Melanoma cells are insensitive to BH3-mimetic drugs as single agents. Extension of data presented in Figure 2 in main text. Selective antagonism of BCL-XL (A1331852), BCL-2 (ABT-199) or MCL-1 (S63845), as well as co-targeting of BCL-XL, BCL-2 and BCL-W (ABT-263) fails to kill the majority of melanoma cell lines unless high concentrations are used. Data represent mean ± standard deviation from n = 3 separate assays.

Supplementary Figure 2. Co-antagonism of several pro-survival BCL-2 proteins has greater effect on cell viability than targeting each protein alone. Extension of data presented in Fig. S3a. Antagonism of MCL-1 plus BCL-XL (S63845 + either A1331852 or ABT-263) is more effective than the combination targeting MCL-1 plus BCL-2 (S63845 + ABT-199) in all melanoma cell lines tested. Cell viability was determined after 24 h treatment by CellTiter-Glo luminescent assay. Data represent mean ± standard deviation from n = 3-4 separate assays.

Supplementary Figure 3. Drug combinations targeting MCL-1 plus BCL-XL or BCL-2 act synergistically. Synergy analysis was performed for BH3-mimetic combinations on **a** established and

b patient-derived cell lines using Combenefit software (36). Data presented were produced using the Bliss model, however, nearly identical outcomes were produced using the Loewe and Highest Single Agent models.

Supplementary Figure 4. Treatment with BH3-mimetic drugs for 72 h has causes only a minor increase in melanoma cell killing compared to 24 h treatment. **a** Cells were treated with S63845, ABT-263 and various combinations for 72 h before analysis by CellTiter-Glo viability assay. **b** EC_{50} values for drug combinations at 24 (from Supplementary Table 1) and 72 h. Data represent mean ± standard deviation from n = 3 separate assays.

Supplementary Figure 5: BH3-mimetics combinations are synergistic in 3D cultures. A02 cells were allowed to form spheroids over 72 h, then spheroids embedded in collagen matrix and treated for 72 h with drugs or combinations as indicated. Bright field images (left panel) and fluorescence images for DRAQ7 staining (right panel) are shown for each combination. The panel in the top right (red box) represents the vehicle only control. Spheroids were imaged with an Olympus FV3000 Laser Scanning Confocal Microscope/Olympus UPLSAPO 4×. Note differences in DRAQ7 intensity and distribution. Data are representative of N = 2 independent experiments with three spheroids per condition.

Supplementary Figure 6. BAX and BAK deletion reduce sensitivity of melanoma cell lines to BH3-mimetic drug combinations. **a** Doxycycline-induced expression of sgRNAs targeting *BAK* or *BAX* in Cas9-expressing LM-MEL-28 melanoma cells results in a significant reduction in BAK or BAX protein levels, respectively, as determined by Western blotting. Probing for β-actin was used as a loading control. **b** Although deficiency (dotted lines) in BAK (left panel) or BAX (right panel) did not significantly impact sensitivity to single-agent treatment with BH3-mimetic drugs (S63845, ABT-

263, ABT-199, A1331852), cells lacking BAK or BAX were more resistant to combination BH3-mimetic drug treatments than wild-type cells.

Supplementary Figure 7. Deletion of BFL-1 together with antagonism of other pro-survival BCL-2 family members has only minor impact on melanoma cell survival. **a** CRISPR/Cas9-mediated deletion BFL-1 in M14 and LM-MEL-28 melanoma cell lines confirmed by Western blot analysis. **b** EC_{50} values for BH3-mimetic combinations in each BFL-1-deleted (*BCL2A1* sgRNA) and control (*BCL2A1* wt) cell line. Data represent mean ± standard deviation from n = 3 separate assays.

Supplementary Figure 8. Treatment of melanoma cell lines with bortezomib induces NOXA protein expression at concentrations where enhanced cell killing effects with S63845 were also observed, as determined by Western blotting. Probing for β-actin was used as a loading control.

	BRAF	NRAS
Established lines		
CHL-1	WT	WT
MeWo	WT	WT
A375	V600E	WT
A02	V600E	WT
M14	V600E	WT
UACC 257	V600E	WT
SK-MEL-5	V600E	WT
Patient-derived lines		
LM-MEL-28	V600E	WT
LM-MEL-33	V600E	WT
LM-MEL-34	WT	Q61Q
LM-MEL-53	WT	WT

Supplementary Table 2

	EC_{50} values (nM)												
Cell line	S63845		S63845 + ABT-263				S63845 + ABT-199				S63845 + A1331852		
	-	ABT-263	5 µM ABT-263	0.5 µM ABT-263	0.05 µM ABT-263	ABT-199	5 µM ABT-199	0.5 µM ABT-199	0.05 µM ABT-199	A1331852	5 µM A1331852	0.5 µM A1331852	0.05 µM A1331852
CHL-1	9 490	6 760	1.66	13.6	208	5 840	30.4	347	604	14 100	2.96	38.6	174
MeWo	6 820	12 000	16.6	115	438	5 560	61.7	518	676	21 400	30.3	191	373
A375	13 200	8 910	5.88	55.7	1 230	17 400	364	7 080	11 500	10 500	15.4	35.2	411
A02	8 350	11 000	4.32	34.9	279	16 200	16.2	552	1 840	20 000	17.6	187	555
M14	13 100	10 470	89.0	1 460	4 240	14 800	1 230	5 890	11 500	23 400	631	1 820	3 470
UACC 257	4 300	21 900	4.81	35.3	121	22 400	14.8	253	1 700	26 900	3.48	22.7	130
SK-MEL-5	13 200	12 300	1.79	204	4 680	10 700	186	9 550	11 200	16 600	20.6	127	2 890
LM-MEL-28	7 480	5 750	8.24	60.5	434	12 000	42.8	1 540	4 220	15 100	22.1	100	613
LM-MEL-33	8 180	12 900	6.26	67.4	821	15 100	219	2 740	7 530	13 800	8.48	37.7	365
LM-MEL-34	1 340	3 550	0.472	3.61	15.1	11 200	1.40	44.3	159	7 410	1.50	4.67	14.7
LM-MEL-53	9 150	5 890	1.22	1.82	53.5	12 900	10.4	2 480	11 200	1 000	1.47	1.61	1.75

Supplementary Figure 1

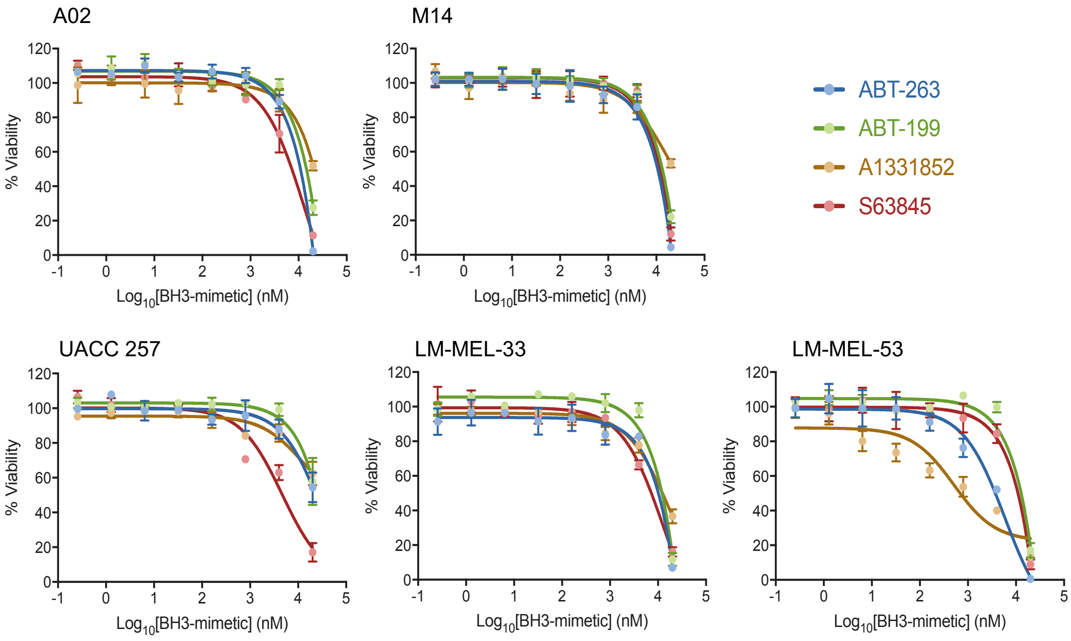

Supplementary Figure 2

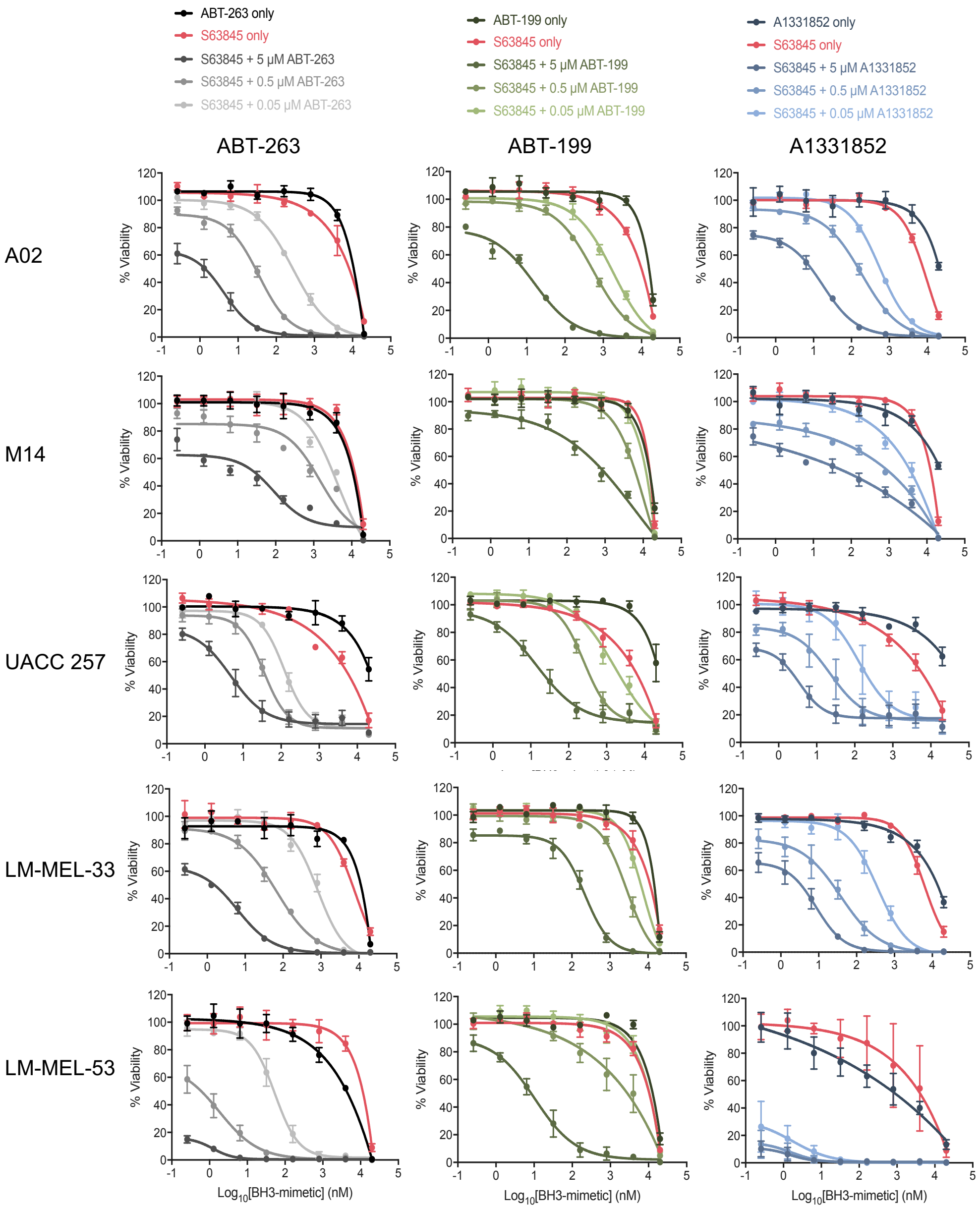

Supplementary Figure 3

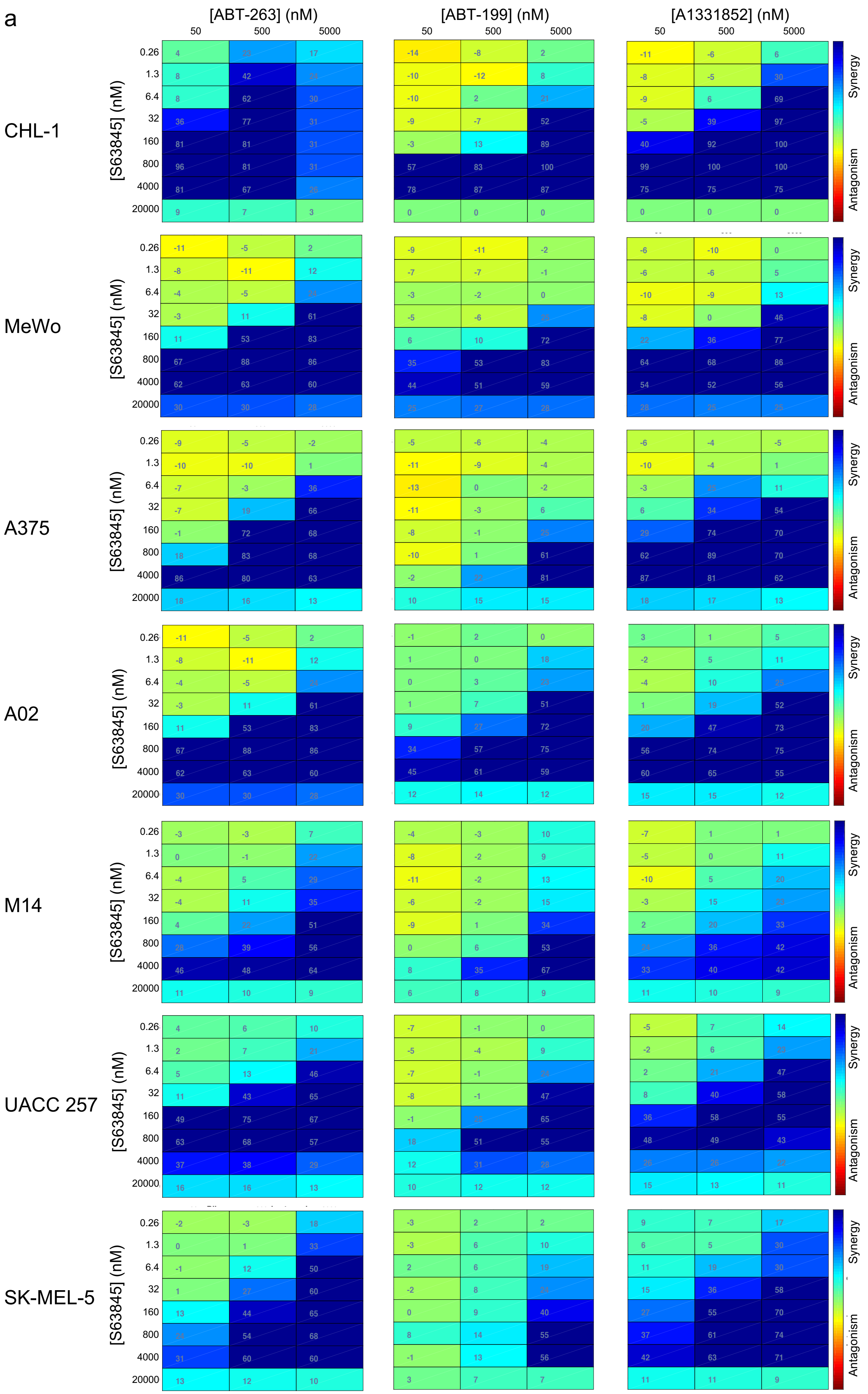

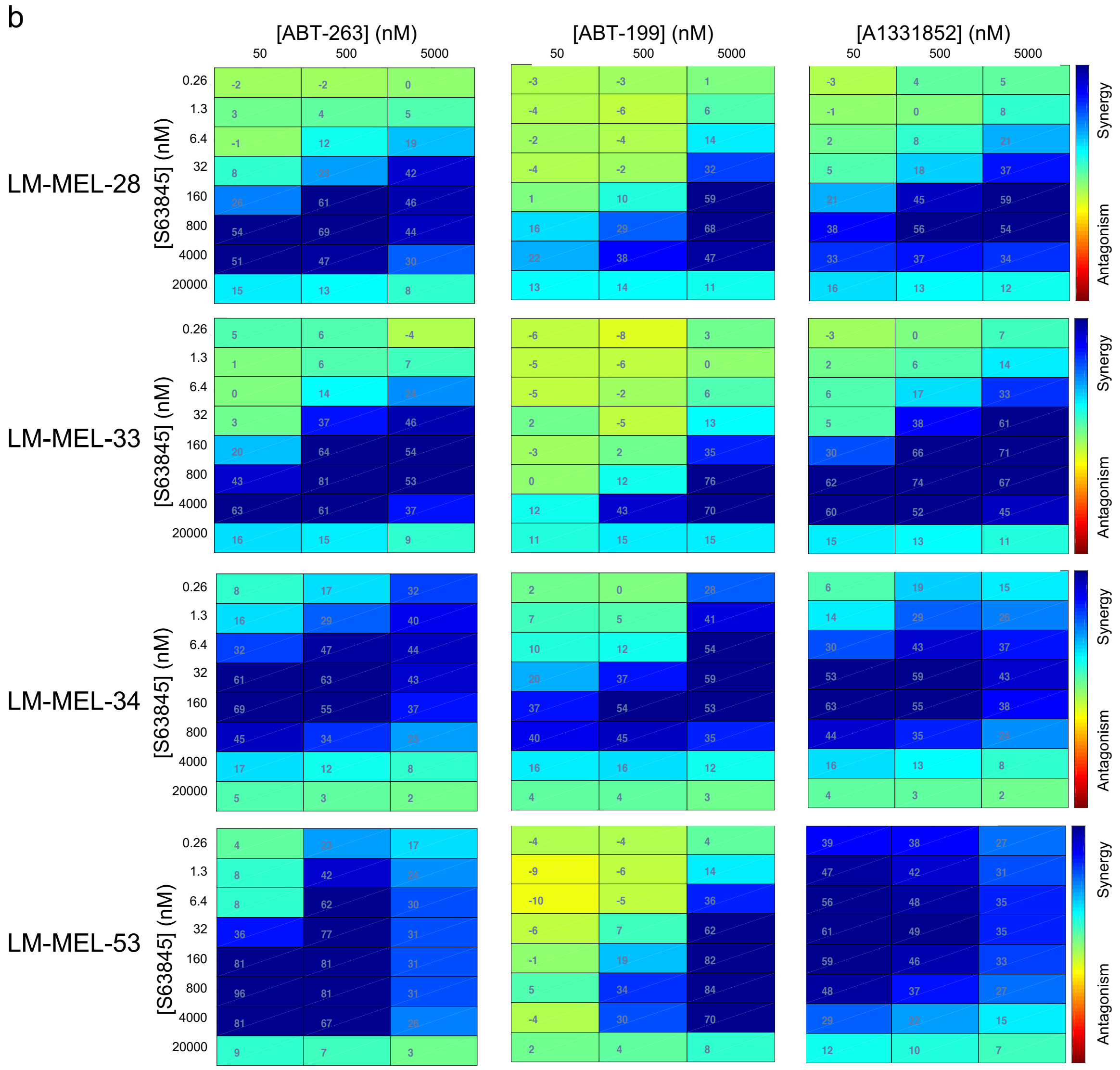
b
[ABT-263] (nM)
[ABT-199] (nM)
[A1331852] (nM)
50
500
5000
[S63845] (nM)
0.26
1.3
6.4
32
160
800
4000
20000
LM-MEL-28
LM-MEL-33
LM-MEL-34
LM-MEL-53
Synergy
Antagonism

a

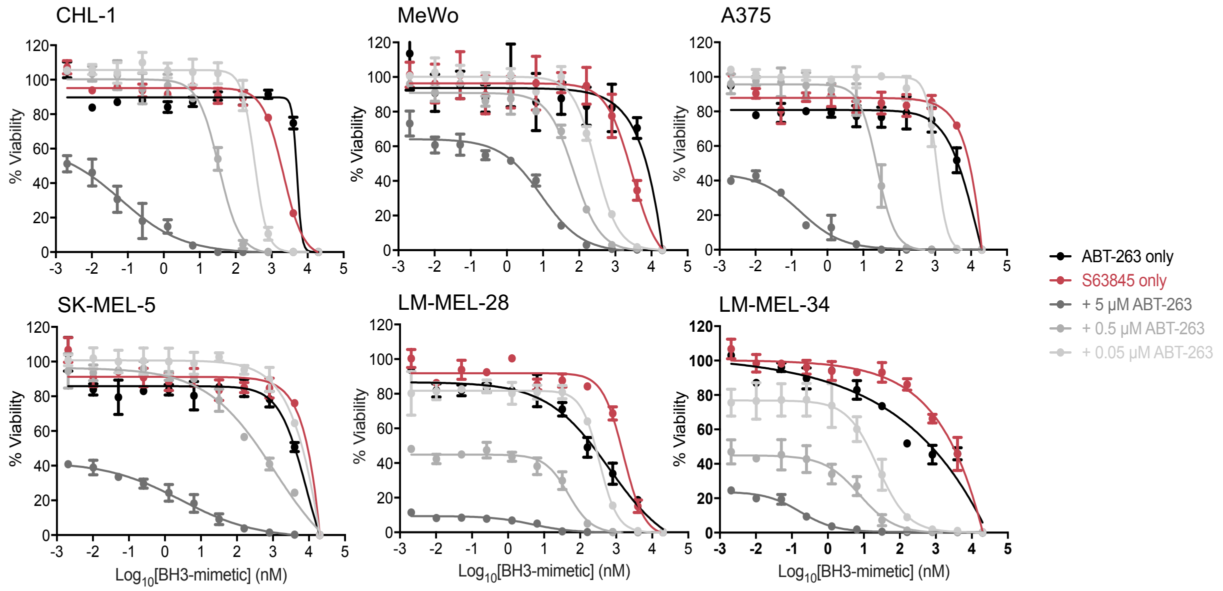

b

	EC50 values (nM)									
	S63845		-		S63845 + ABT-263					
	-		ABT-263		5 μM ABT-263		0.5 μM ABT-263		0.05 μM ABT-263	
	24 h	72 h	24 h	72 h	24 h	72 h	24 h	72 h	24 h	72 h
CHL-1	9 490	2 080	6 760	5 080	1.66	0.0638	13.6	34.0	208	333
MeWo	6 820	2 970	12 000	8 320	16.6	9.02	115	71.6	438	286
A375	13 200	11 000	8 910	6 610	5.88	0.175	55.7	24.4	1 230	1 110
SK-MEL-5	13 200	11 200	12 300	8 590	1.79	2.82	204	1 120	4 680	6 610
LM-MEL-28	7 480	1 590	5 750	648	8.24	4.77	60.5	46.8	434	342
LM-MEL-34	1 340	3 240	3 550	776	0.472	0.172	3.61	7.46	15.1	24.1

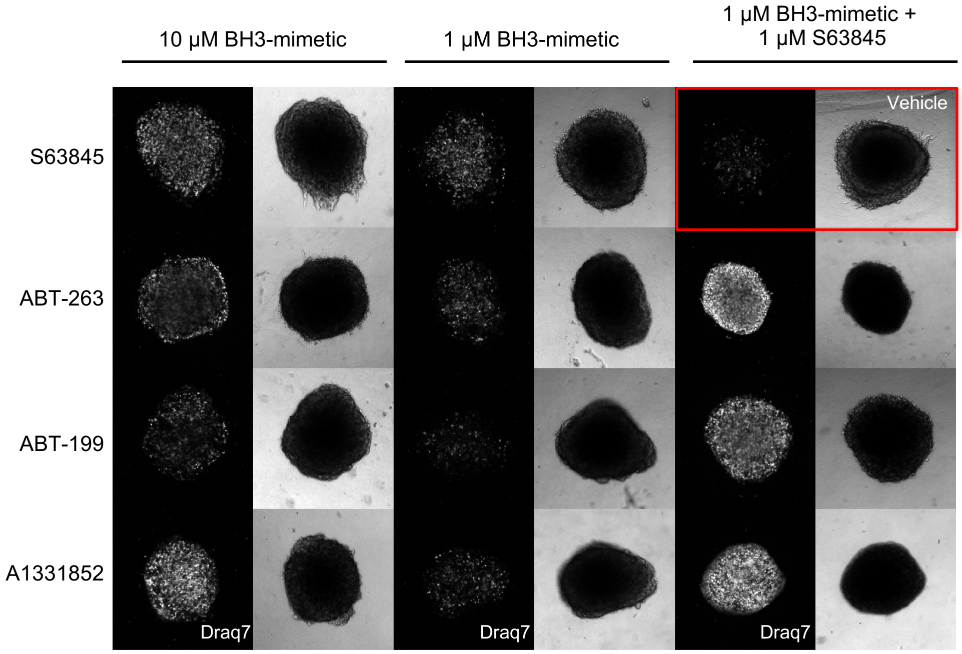
10 µM BH3-mimetic
1 µM BH3-mimetic
1 µM BH3-mimetic +
1 µM S63845
S63845
ABT-263
ABT-199
A1331852
Vehicle
Draq7
Draq7
Draq7

Supplementary Figure 6

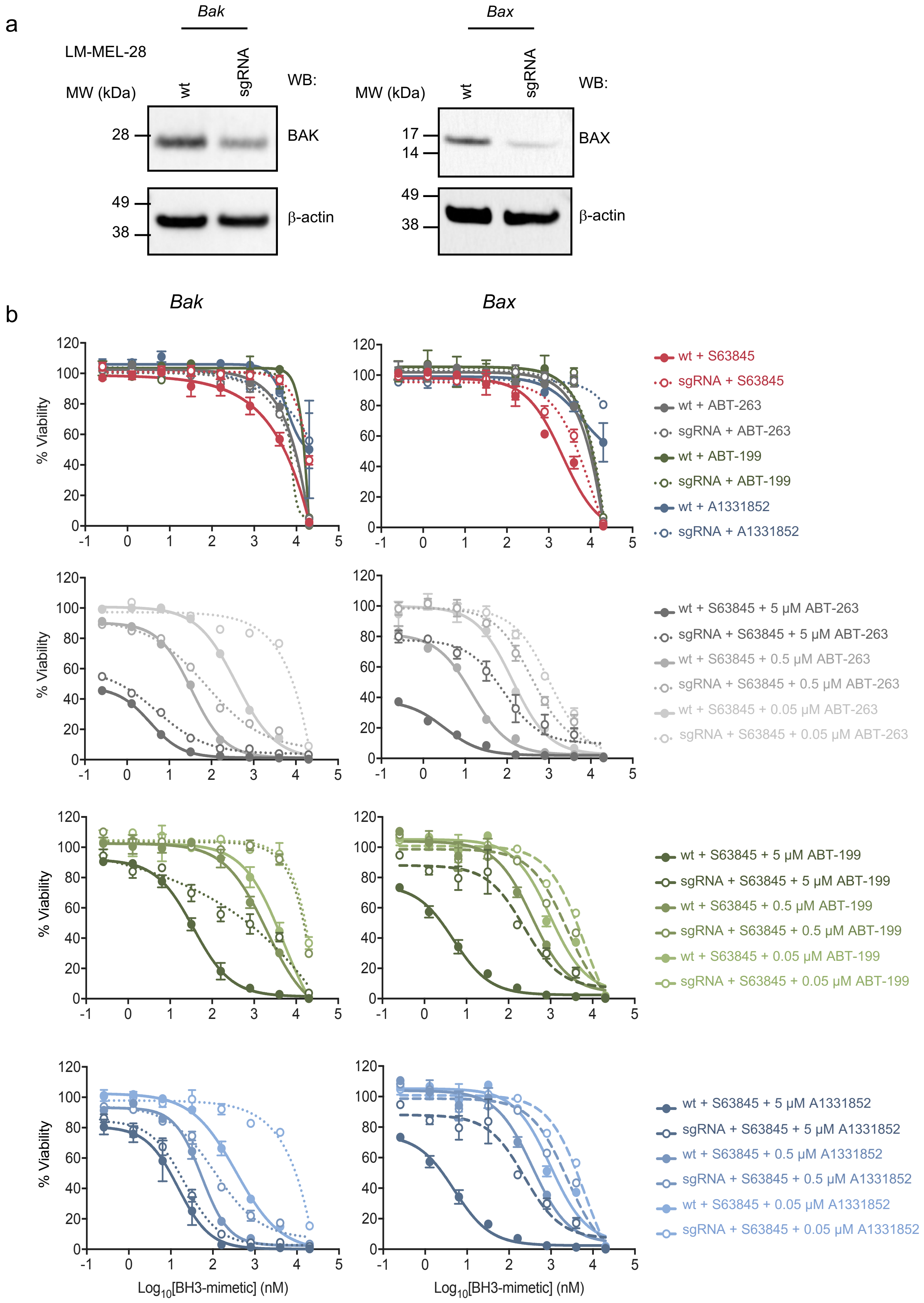

a

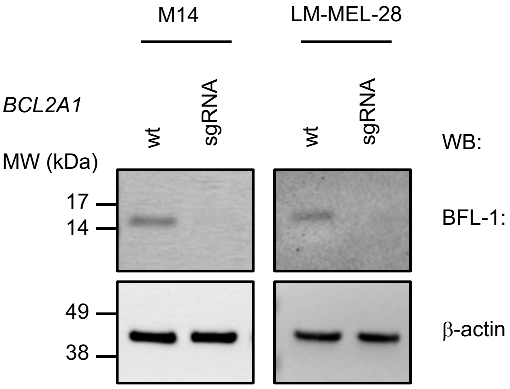

b

Cell line:		EC$_{50}$ values (nM)			
		M14		LM-MEL-28	
		BCL2A1 wt	*BCL2A1* sgRNA	*BCL2A1* wt	*BCL2A1* sgRNA
S63845	-	6 550	2 830	2 500	829
	ABT-263	17 000	12 600	8 510	10 200
S63845 + ABT-263	5 µM ABT-263	221	33.9	17.0	9.72
	0.5 µM ABT-263	1 580	268	109	59.9
	0.05 µM ABT-263	2 630	919	508	210
	ABT-199	7 080	2 450	2 830	854
S63845 + ABT-199	5 µM ABT-199	686	255	83.2	54.6
	0.5 µM ABT-199	3 350	1 150	836	350
	0.05 µM ABT-199	5 570	1 830	975	490
	A1331852	20 000	20 000	18 600	13 200
S63845 + A1331852	5 µM A1331852	238	63.9	53.1	32.7
	0.5 µM A1331852	1 240	349	188	91.5
	0.05 µM A1331852	1 540	633	558	218

Supplementary Figure 8

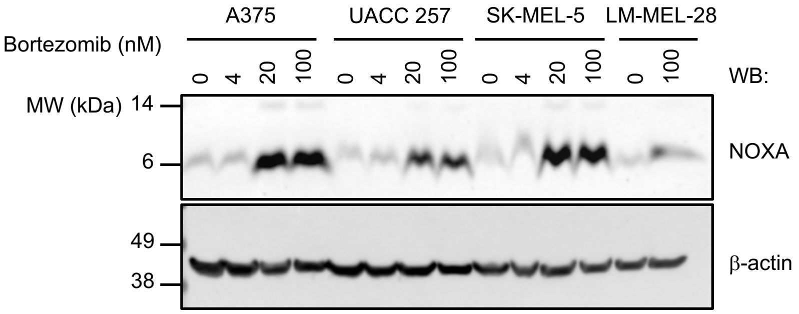

Supplement: Supplementary file 1 — Supplementary Material [file 41419_2019_1568_MOESM1_ESM.pdf]
